# Operational and geological controls of coupled poroelastic stressing and pore-pressure accumulation along faults: Induced earthquakes in Pohang, South Korea

**DOI:** 10.1038/s41598-020-58881-z

**Published:** 2020-02-07

**Authors:** Kyung Won Chang, Hongkyu Yoon, YoungHee Kim, Moo Yul Lee

**Affiliations:** 1 Sandia National Laboratories, Geotechnology & Engineering Department, Albuquerque, 87105 USA; 2 Sandia National Laboratories, Geomechanics Department, Albuquerque, 87105 USA; 3 Seoul National University, School of Earth and Environmental Sciences, Seoul, South Korea

**Keywords:** Hydrology, Solid Earth sciences

## Abstract

Coupled poroelastic stressing and pore-pressure accumulation along pre-existing faults in deep basement contribute to recent occurrence of seismic events at subsurface energy exploration sites. Our coupled fluid-flow and geomechanical model describes the physical processes inducing seismicity corresponding to the sequential stimulation operations in Pohang, South Korea. Simulation results show that prolonged accumulation of poroelastic energy and pore pressure along a fault can nucleate seismic events larger than *M*_w_3 even after terminating well operations. In particular the possibility of large seismic events can be increased by multiple-well operations with alternate injection and extraction that can enhance the degree of pore-pressure diffusion and subsequent stress transfer through a rigid and low-permeability rock to the fault. This study demonstrates that the proper mechanistic model and optimal well operations need to be accounted for to mitigate unexpected seismic hazards in the presence of the site-specific uncertainty such as hidden/undetected faults and stress regime.

## Introduction

Over the past decade elevated levels of seismic activities have been observed at the sites related to subsurface energy exploration activities, including wastewater injection for conventional and unconventional oil/gas development^1–4^ and geothermal stimulation^5–7^. Despite the recent progress in statistical and physics-based investigation of induced seismicity^8–10^, recent unexpected moderate to large magnitude earthquakes (${M}_{{\rm{w}}}\ge 3$) after shut-in (e.g., 2006 *M*_w_3.2 Basel, Switzerland^11^, 2017 *M*_w_5.5 Pohang, South Korea^7^) show the need of the mechanistic study to understand underlying physical mechanisms. Since fluid injection-extraction associated with these elevated earthquakes has often been operated with multiple wells, interactions of well operations and other hydrogeological features need to be further investigated.

Large earthquakes require large seismogenic faults, and pressure perturbation and shear stressing are two primary factors to trigger fault slip by reducing fault strength^12–14^. Pore-pressure accumulation along conductive faults has been considered as the principal mechanism for inducing seismicity^3,15^ in which diffusive propagation of pressure plumes is essential, but controlled by hydraulic connectivity from faults to the fluid-injection reservoir. Another primary mechanism is the poroelastic stressing in which the volumetric changes of the pressurized zone perturb the stress field of the surrounding rock by transmitting elastic forces to longer distances even beyond the hydraulically affected region such as distant and disconnected basement faults^16–19^. Temporal changes in stress states at frictional faults will determine the onset of fault slip corresponding to rate-and-state friction mechanisms^20,21^ that can generate delayed surge of seismic events along faults even after shut-in.

Site-specific features of geological formation and/or operational controls govern spatio-temporal patterns, rates, and magnitudes of induced seismicity observed at subsurface energy exploration sites^3,9,10,22–24^. However, some induced seismicity events were observed along the hidden or not well-defined faults (e.g., Pohang, South Korea^7,25,26^), addressing that interpretation of seismic activities prior to a large earthquake requires a more comprehensive mechanistic model to properly characterize the faults and surrounding formation. Also, multiple well operations are common to stimulate a subsurface system or dispose a volume of wastewater, but the geomechanical influence of injection-extraction operations through multiple wells on adjacent faulting system are not throughly investigated. Recent experimental results including direct fluid injection into a natural fault^27^ and U.S. DOE (Department of Energy) geothermal stimulation activities in the Sanford underground testing facility (the EGS Collab, https://eesa.lbl.gov/projects/the-egs-collab-project/) reveal that aseismic processes modeled by a rate-dependent friction law can be used to identify a precursor to seismic slip^27^ and the locations of seismic events can be directly monitored to delineate creation of a hydraulic fracture and additional reactivation of pre-existing structures. However, induced-seismicity hazards are still being statistically modeled, which typically relies on empirical analyses of the observed rate of seismic events on a per-well basis and physics-based models to account for site-specific geological and operational constraints are rarely used.

**Figure 1:**
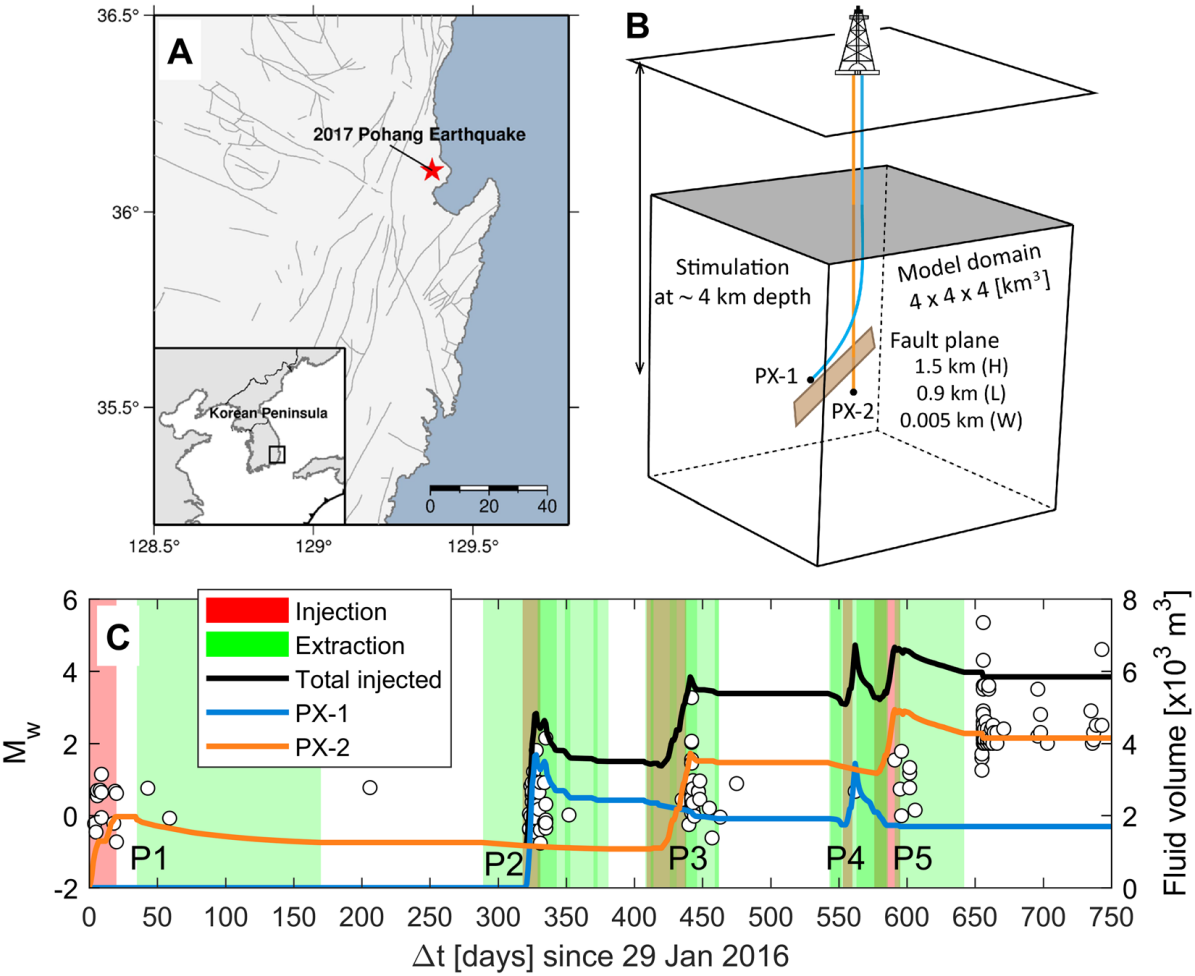
(**A**) Map showing the epicenter of the 2017 Pohang earthquake. Inset map shows tectonic setting of Korean Peninsula. Gray lines represent fault locations. Map was produced using Generic Mapping Tools (GMT) software, version GMT 4.5.9^54^ (http://gmt.soest.hawaii.edu/projects/gmt). (**B**) Schematic description of the numerical domain consists of the basement rock and a main fault plane. (**C**) Sequential distribution of well operations and observed seismic events with magnitude at the Pohang EGS site^26,28,42^. The accumulative injection volume is indicated by a black solid line. A total of five stimulation phases are named as P1, P3, P5 (PX-2 well) and P2 & P4 (PX-1 well) in the order of stimulations and the accumulative injection volume for each well is indicated by blue and orange lines, respectively.

In this study, we elaborate the following critical questions; (1) what are the physical mechanisms inducing moderate to large earthquakes (${M}_{{\rm{w}}}\ge 3$) after pausing/terminating well operations? and (2) how do geological and operational parameters affect spatio-temporal patterns of seismic events? We examine the poroelastic response of the basement fault to sequential stimulation operations that were motivated at the Pohang ehanced geothermal system (EGS) site using a three-dimensional (3-D) coupled simulation with mechanistic analyses (Figs. 1 and S1 and Table 1). Impacts of pore-pressure accumulation and poroelastic stressing are quantified by analyzing the Coulomb stress changes that are correlated with spatio-temporal distribution of observed seismic events. Additional simulations with various geological and operational factors will emphasize the importance of physical characterization of faults and surrounding basement and adequate injection-extraction operations to mitigate the risk of induced seismicity prior to and/or during subsurface energy activities.

**Table 1:** Hydrological and mechanical parameters for the reference model*.

	Poroelastic and transport properties^†^		
	Basement (*b*)	Fault (*f*)	Fluid (*w*)
*κ*_*i*_ (m^2^)	7.6 × 10^−19^	1 × 10^−15^	—
${\phi }_{i}$ (−)	0.0048	0.02	—
${G}_{i}$ (GPa)	13.8^‡^	6	—
${\lambda }_{i}$ (GPa)	10^‡^	4	—
${\lambda }_{u,i}$ (GPa)	20.8	11.6	—
${\nu }_{i}$ (−)	0.21^‡^	0.2	—
${\alpha }_{i}$ (−)	0.4	0.79	—
${\rho }_{i}$ (kg/m^3^)	2740	2500	1000
$\eta$ (Pa$\cdot$s)	—	—	0.4 × 10^−3§^
*D*_*i*_ (m^2^/s)	1 × 10^−4^	8.3 × 10^−3^	—

*The parameter values are for the reference Case 1 in Table 2.

^†^The subscript *i* represents each material used in the model: basement (*b*), fault (*f*), and fluid (*w*).

^‡^Mechanical parameter values of basement rocks for a drained condition are from^28^.

^§^Brine viscosity ranges 0.4 × 10^−3^(±0.05 × 10^−3^)^55^.

## Results

### Pore-pressure accumulation and poroelastic stressing

**Figure 2:**
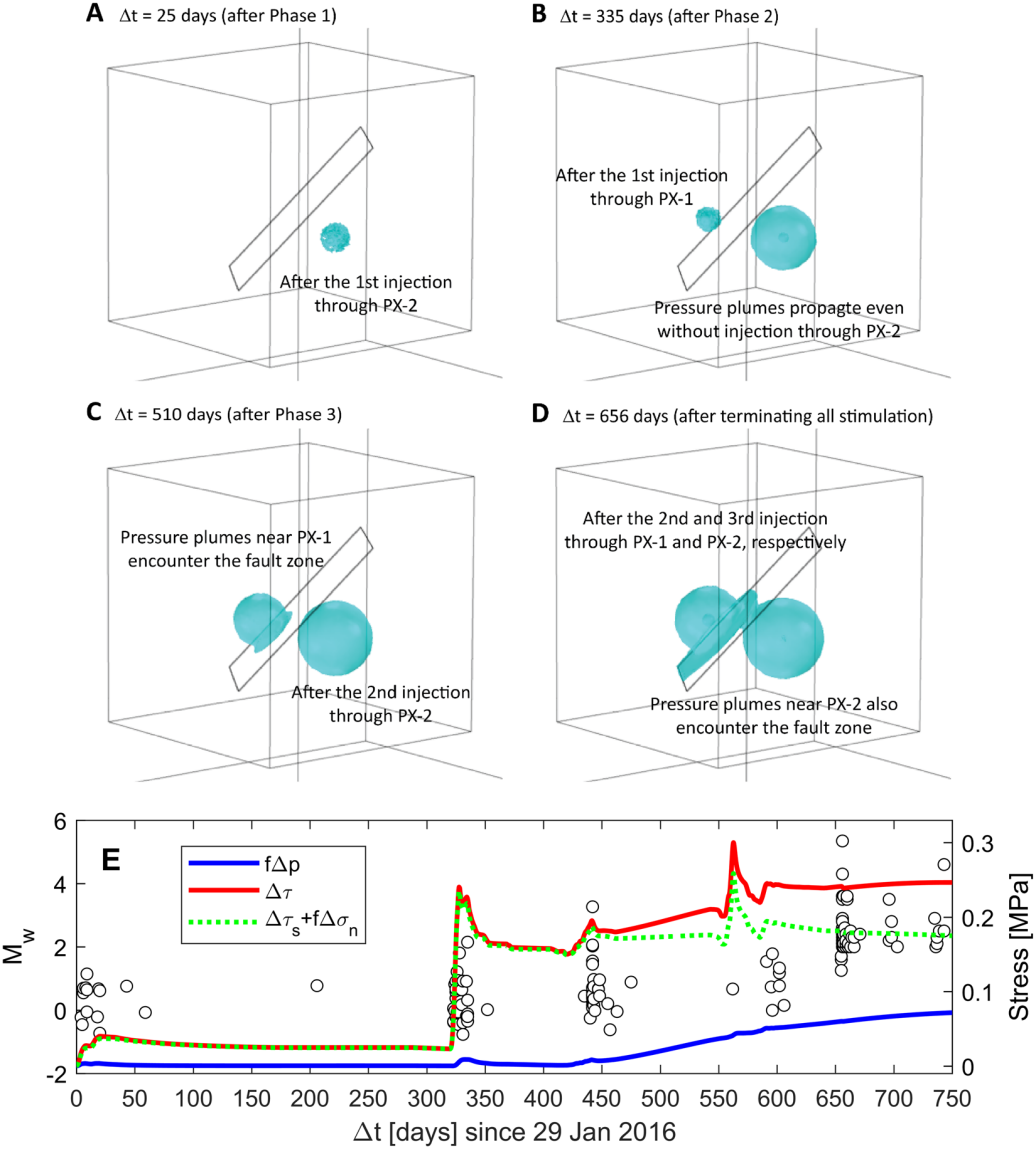
Simulation results are described after each stimulation phase at different times. (**A–D**) The 3-D spatial distribution of the isosurface at the level of *f*Δ*p* = 0.01 MPa. (**E**) Comparison of the observed *M*_w_ trend with components of the Coulomb stress change (Δ*τ*); *f*Δ*p* quantifies direct impact of pore-pressure diffusion whereas $\Delta {\tau }_{s}+f\Delta {\sigma }_{n}$ represents poroelastic stressing.

The coupled poroelasticity model was used to simulate pore pressure and stress changes due to fluid injection-extraction operations at Pohang as shown in Fig. 1. All parameter values are listed in Table 1. The 3-D spatial distributions of pore-pressure isosurface at the level of $f\Delta p=0.01$ MPa (Fig. 2A–D) show the expansion of pressurized regions over time due to geothermal stimulations at two wells (PX-1 and PX-2; Fig. 1C). The low-permeability basement rock inhibits considerable pressure perturbation on the fault even after completing Phases 1 and 2 stimulation activities (Fig. 2A,B). The pressurized region grown steadily from PX-1 stimulations encounters the fault zone after Phase 3 stimulation (Fig. 2C). Subsequent injection-extraction activities at Phases 4 and 5 lead to intervention of pressure plumes initiated from PX-2 afterwards, and then high-permeability fault allows rapid diffusion of pore pressure throughout the fault zone (Fig. 2D). The low permeability of the surrounding basement rock limits diffusion of pore pressure across the lithological boundary, and thus accumulated pore pressure within the fault zone prefers to spread parallel to the fault plane.

Comparison of the observed seismic moment magnitude (*M*_w_) trend with temporal evolutions of the Coulomb stress components ($f\Delta p$ and $\Delta {\tau }_{s}+f\Delta {\sigma }_{n}$) at the hypocenter within the fault plane (Fig. 2E) suggests that two physical processes control temporal sequences of seismic events along the permeable fault; $f\Delta p$ represents the direct effect of pore-pressure diffusion into the fault whereas $\Delta {\tau }_{s}+f\Delta {\sigma }_{n}$ quantifies the poroelastic stress transfer through the basement rock to the fault. Figure 2E shows that rapid poroelastic response to stimulation phases causes $\Delta {\tau }_{s}+f\Delta {\sigma }_{n}$ to increase immediately. The trend of poroelastic responses matches the induced seismic events very well during and after Phases 1 to 3. Once pore pressure diffuses into the fault, gradual increases in $f\Delta p$ (trapped by low-permeability basement) reduces the normal load acting on the fault plane. Relatively short-term injection through PX-1 at Phase 4 causes substantial poroelastic stressing, but following extraction through both PX-1 and PX-2 releases elastic energy promptly that mitigates earthquake nucleation. On the other hand, injection and subsequent extraction only through PX-2 at Phase 5 may not attenuate poroelastic stresses and pore pressure in the fault sufficiently, inducing a number of seismic events less than *M*_w_2. Most seismic events larger than *M*_w_3 at the Pohang site were observed after terminating stimulation activities. This observation suggest that the enlargement of pressurized regions within the fault plane as shown in simulations results (Fig. 2A–D) can continuously influence shearing and accumulation of pore pressure along the fault, which can lead to larger Δ*τ*, thereby causing more substantial aftershocks.

### Coulomb stress changes along a fault

**Figure 3:**
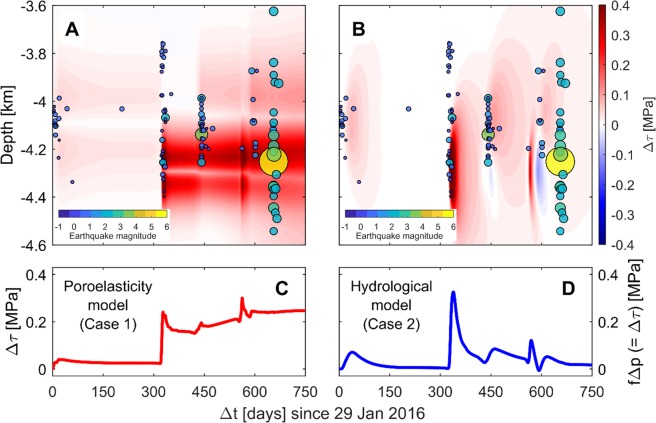
Spatio-temporal fitting of the Pohang seismic events to the Coulomb stress change (Δ*τ*) along the fault plane. (**A,B**) The distribution of Δ*τ* over depths along the middle of the fault zone and the observed seismic events over time. The magnitude of earthquakes varies with size and color of circles. (**C,D**) The evolution of Δ*τ* at the hypocenter from the poroelasticity (coupled) model and the hydrological (uncoupled) model.

Coulomb stress can be perturbed locally by stimulation activities which can lead to nucleate seismic events and cause large-magnitude seismic event after shut-in. For the reference case (Case 1; see Table 2 for key parameter values), the distribution of the total Coulomb stress change (Δ*τ*) over time was compared with the spatial patterns of observed seismic events over the depth of the fault (−3.6 to −4.6 km deep) in Fig. 3A. Although actual seismic events occurred in different lateral locations^28^, we compared Δ*τ* along the central vertical line of the fault with the observed seismic events at the same depth since Δ*τ* from simulation results is likely to be higher along the central vertical line of the fault.

**Table 2:** Variation in well operation and formation properties for sensitivity tests^*^.

Case	Coupling	Well number	Basement	Fault		Figure
			*κ*_*b*_ (m^2^)	*κ*_*f*_^†^ (m^2^)	*G*_*f*_^‡^ (GPa)	
1	Yes	PX-1/PX-2	7.6 × 10^−19^	1.0 × 10^−15^	6	2C, 3A,C
2^§^	No	—	7.6 × 10^−17^	—	—	3B,D
3	—	PX-2	-	—	—	4C
4	—	—	7.6 × 10^−17^	—	—	4D
5	—	—	—	1.0 × 10^−12^	—	4E
6	—	—	—	—	20	4F

^*^The empty cell indicated by the hyphen has the same value used in the reference Case 1.

^†^The fault permeability varies depending on its internal architecture, ranging from 10^−12^ to 10^−22^ m^2^ ^53^.

^‡^The modulus of rigidity for fault damage zone is highly variable, and estimated to range from 0.8 to 20 GPa (converted from Young’s modulus (*E*) and Poisson’s ratio ($\nu$) values given in^43^).

^§^The uncoupled model with larger permeability (converted to ${D}_{b}=1\times {10}^{-2}$ m^2^/s) resembles the hydrological model used in^28^.

As presented above with Fig. 2E, shear stressing on the fault plane can induce instantaneous seismic events due to poroelastic response to the stimulation Phases 1 to 3 before direct pore-pressure effects on the fault plane become important. Spatio-temporal comparison between the coupled model results and seismic events in Fig. 3A shows that the overall trend of seismic events during the Phases 1 to 3 period qualitatively matches the simulated stress change relatively well. The fact that the simulated Δ*τ* tends to propagate through the bottom half of the fault plane may suggest that some observed seismic activities in the upper part of the fault plane (e.g., a cluster at the depth of ~3.8 to 3.9 km at $\Delta t\approx 335$ days after Phase 2 injection through PX-1) could be associated with other processes that are not accounted for in this work, such as hydraulic fracturing or reopening preexisting fractures away from the seismogenic fault plane. The seismic cluster after Phase 3 was confined within shorter depths compared to the cluster after Phase 2. This observation would suggest that poroelastic stressing driven by injection through PX-2 at Phase 3 localizes the seismic events along the fault.

Once pore pressure diffuses into the fault zone ($\Delta t\ge 450$ days; refer to Fig. 2C), either instantaneous poroelastic stressing and/or increase in pore pressure can be primary mechanisms for weakening the fault plane. At this stage, the prolonged diffusion of pore pressure can have a broader impact on Δ*τ* over the larger distance along the fault. However, injection-extraction through PX-1 and/or PX-2 at Phases 4 and 5 attenuated or enhanced the poroelastic stressing effect, which could be critical to prevent or initiate earthquake nucleation. After shut-in, continuous accumulation of poroelastic strain energy and pore pressure directly diffused from both sides of the fault can be sufficient to generate moderate to large magnitude earthquake along the fault, which may be a primary or one of reasons for the *M*_w_5.5 earthquake observed at the depth of ~4.27 km^28^.

The hydrological model (Case 2) implements two orders of magnitude higher permeability for basement which is the practical upper limit of the estimated values from hydraulic modeling calibration and analytical Jacob method^28^. Under a diffusion-dominant system, the pore-pressure buildup within the permeable fault is essential to nucleate large-magnitude earthquakes, which is controlled by a contrast of hydraulic diffusivity between the fault and bounding basement. Rapid hydraulic response to stimulation activities captures intermittent seismic event, and a sharp increase in pore pressure after stimulation Phase 2 (injection through PX-1) may account for a burst of seismic activity around $\Delta t\approx 335$ days (Figs. 3B and S2). However, post shut-in moderate to large seismic events, including *M*_w_5.5 event, are unlikely to occur due to substantial dissipation of elevated pore pressure into the high-diffusivity basement after extraction or shut-in. Similar behavior of pore pressure within the fault is also observed in the previous hydrological model^28^ (refer to Fig. S3).

The $\Delta \tau (t)$ trends at the hypocenter distinguish the dominant mechanisms inducing seismicity for the coupled from uncoupled systems. For the coupled model (Case 1) the steep changes in Δ*τ* reflect the poroelastic stressing corresponding to injection-extraction operations and the gradual increase in Δ*τ* reflects pore-pressure diffusion through the low permeable basement rock. As a result of the coupling effect, $\Delta \tau$ increases by ~0.25 MPa even after all stimulations (Fig. 3C), which can give us a mechanistic insight of the elevated seismic events larger than *M*_w_3 at the Pohang EGS site. However, for the uncoupled hydrological model (Case 2) cyclic perturbations with quick diminishing trend following the extraction and/or shut-in in Δ*τ* characterizes the diffusion-dominant sequence of $\Delta \tau (\,=\,f\Delta p)$ in Fig. 3D. As a result, the uncoupled model does not predict the Δ*τ* trend correctly and can’t explain the mechanisms of induced seismic events after shut-in.

### Operational constraints

**Figure 4:**
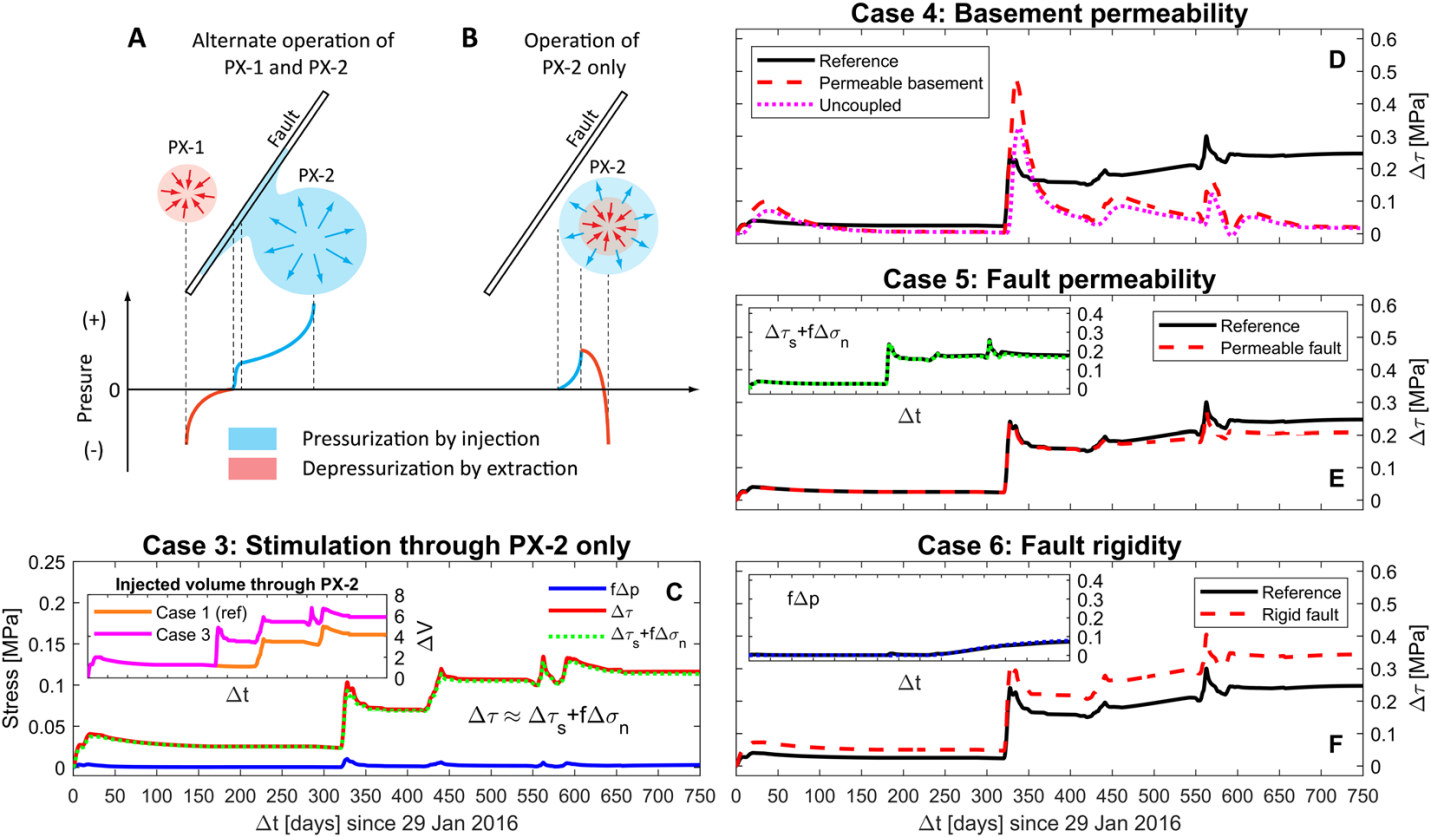
Sensitivity tests for operational and geological parameters. (**A,B**) Schematic description of enhancement mechanisms for two operational scenarios: alternate injection-extraction through PX-1 and PX-2 (Case 1) and cyclic injection-extraction through PX-2 only (Case 3). (**C**) Temporal distribution of *f*Δ*p*, $\Delta {\tau }_{s}+f\Delta {\sigma }_{n}$, and Δ*τ* from Case 3. Subset plot show the total volume of fluid injected through PX-2: orange line for Case 1 (presented in Fig. 1C) whereas magenta line for Case 3. **(D–F**) Effect of basement permeability (${\kappa }_{b}$), fault permeability (${\kappa }_{f}$) and fault rigidity (*G*_*f*_) on the trend of Δ*τ* at the hypocenter. Subset plots in (**E**,**F**) show $\Delta {\tau }_{s}+f\Delta {\sigma }_{n}$ and *f*Δ*p*, respectively.

Stimulation operations with alternate injection-extraction through PX-1 and PX-2 (Case 1; refer to Fig. 1C) can prompt strong gradients in pore-pressure fields across the fault plane that will increase the propagation of fluid pressure and stresses through low-permeability basement rocks (Fig. 4A). Alternative stimulation activities in both PX-1 and PX-2 wells may enhance gradual accumulation of energy on the fault after Phase 3 activities ($\Delta t\ge 450$ days) when pore pressure front was simulated to reach the fault plane.

To clarify the enhancement mechanism by the multiple-well operation, the same injection-extraction scenario through a single well is modeled in which the total volume of fluid is injected through PX-2 only (Case 3). A larger accumulative volume of fluid, indicated by a magenta line in subset plot of Fig. 4C, may lead to substantial enlargement of the pressurized region around PX-2. However, extraction through the same well diminishes gradients across the fault (Fig. 4B), which limits the extent of pressurized region. Thus, no substantial elevation of pore pressure is observed at the fault over time ($\Delta \tau \simeq \Delta {\tau }_{s}+f\Delta {\sigma }_{n}$; Figs. 4C and S4) which may attenuate the direct effect of pore-pressure buildup on induced seismicity. This result emphasizes the need for proper well design and operating strategy with respect to the geometry of preexisting faults to avoid unexpected perturbations in stress states.

### Effect of basement and fault properties

The low-permeability basement rock slows down the diffusion of pore pressure into the fault, but enhance the efficacy of trapping accumulated pore pressure within the fault, consequently increasing Δ*τ* after shut-in (refer to reference Case 1 in Fig. 4D). On the other hand, the more permeable basement (Case 4) develops a diffusion-dominant environment within the fault zone, which generates rapid perturbations in pore pressure and subsequent Coulomb stress as observed from the uncoupled model (Figs. 4D and S5). Note that the presence of high-permeability structures (e.g., fractures) or larger injection rates and longer stimulation periods will enhance the stress transmission and pore-pressure diffusion to the fault, ultimately raising Δ*τ* along the fault.

The fault permeability (Case 5) and rigidity (Case 6) predominantly impact the pore pressure ($f\Delta p$) and the stress components ($\Delta {\tau }_{s}+f\Delta {\sigma }_{n}$), respectively, as shown in Fig. 4E,F. Since the same basement properties are used, the effect of the fault permeability appears after ~450 days while the effect of the fault rigidity appears immediately with Phase 1 stimulation due to the fast elastic stress transfer. A highly permeable fault (larger ${\kappa }_{f}$; Case 5) allows rapid spreading of pore pressure throughout the whole fault plane once pore pressure encounters the fault, so that less $f\Delta p$ is obtained (Fig. 4E). Almost no deviation of $\Delta {\tau }_{s}+f\Delta {\sigma }_{n}$ is observed at more permeable fault because the fault permeability is a primary parameter to control diffusion processes (subset plot in Fig. 4E). Note that the scale of a seismogenic fault zone is another governing factor to determine the extent of pressurized region and the rate of pore-pressure buildup within the fault (i.e., more diffusion is required to generate the same level of $f\Delta p$ along larger faults). A more rigid fault (larger ${G}_{f}$; Case 6) requires more elastic strain energy for slip that generates larger poroelastic stressing on it (Fig. 4F). No significant divergence of $f\Delta p$ implies that fault rigidity controls elastic response to injection-extraction, such that the increase of Δ*τ* in more rigid fault is mainly due to poroelastic stressing (subset plot in Fig. 4F).

## Discussion

### Sequential mechanisms controlling pohang earthquakes

**Figure 5:**
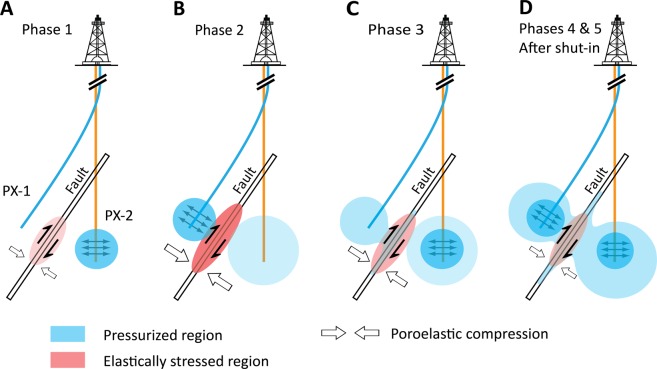
Schematic description of the physical mechanisms for the 2017 Pohang earthquake associated with sequential EGS stimulation activities. (**A**) Phase 1 (the first injection at PX-2): poroelastic compression causes shearing on the fault plane. (**B**) Phase 2 (the first injection at PX-1): the expansion of pressurized regions at each side of the fault causes stronger poroelastic stressing. (**C**) Phase 3 (the second injection at PX-2): fluids injected at PX-1 start to penetrate into the fault due to the vicinity of PX-1 to the fault. (**D**) Phases 4–5 and after shut-in: combined effect of continuous poroelastic stressing and pore-pressure accumulation on earthquake nucleation, consequently inducing moderate to large earthquakes (*M*_w_ ≥ 3).

Comparison of the spatio-temporal patterns of seismic events detected at the Pohang EGS site with our simulation results leads us to develop a conceptual model of the sequential mechanism of seismic events (Fig. 5). Poroelastic shearing initiated as response to injection through PX-2 during Phase 1 (Fig. 5A), which induced a relatively small number of earthquakes less than ${M}_{{\rm{w}}}1.5$ (Fig. 5A). Both pressure buildup caused by injection through PX-1 and continuous expansion of pressurized region near PX-2 compress the fault simultaneously, which generates more intensive shearing of the reverse fault during Phase 2 (Fig. 5B). Direct pore-pressure diffusion started to weaken the fault during and after Phase 3, and poroelastic shearing continues to weaken the fault (Fig. 5C). Intermittent extraction could inhibit earthquake nucleation by rapid release of poroelastic energy (e.g., no sizable earthquakes observed after Phase 4). Incorporated processes of poroelastic stressing and steady pore-pressure buildup accelerate the fault instability after Phase 5 and terminating all stimulation activities (Fig. 5D), which can cause wider distribution of moderate to large earthquakes including ${M}_{{\rm{w}}}5.5$ event at $\Delta t=656$ days. Note that additional mechanisms including thermal stressing, changes in permeability structure, and fracture opening are expected to be prominent close to wells rather than the fault area; hence our coupled model can represent a site-specific feature of the fault and surrounding basement rock reasonably.

### Mitigation of seismic hazards on basement faults

Even though the primary mechanism of induced seismicity caused by fluid injection such as oil and gas extraction and geothermal stimulations are generally well understood, the predictive models are not yet well established to evaluate the potential of seismic hazards from specific operations^10,29,30^. Existing mitigation strategies have used traffic light protocols with staged magnitude-thresholds that aims to reduce pore-pressure perturbation, primarily relying on the observation of preceding moderate-magnitude events^17,29,31,32^. However, adjustment of operational activities in near real-time has failed to avoid inducing a larger earthquake (e.g., 2015 *M*_w_5.0 event in Cushing, Oklahoma, USA^33^). Injection-extraction through multiple wells or reducing total injection volumes could maintain pore-pressure fields below thresholds for slip or limit further propagation of pressure plumes toward pre-existing faults, but not sufficient to eliminate elastic stress accumulation driven by coupled interaction between fluid flow and rock deformation^34^. As demonstrated in Fig. 3, the uncoupled system with only hydrological model failed to predict the steady increase of the Coulomb stress at later stages (after Phase 3) since relatively small amount of new injection with subsequent extraction was performed (see Figs. 1C or 2E). This strongly suggests that mitigation strategies need to include the proper physical mechanisms such as the poroelasticity model in this study so that optimal design and/or operation of wells can be established over a short time period to limit the potential of seismic hazards.

Controlling of injection-extraction operations at multiple wells can prevent substantial accumulation of pore pressure and elastic energy within a seismogenic zone that minimize the risk of induced seismicity. However, injection-extraction at multiple wells near existing and/or newly observed faults can generate substantial hydraulic gradients along or across the faults that control the direction and amount of energy transferred through rigid and low-permeability basement rocks to the faults. Comparison of Cases 1 and 3 in this study (Fig. 2E vs 4C) shows that multiple well operations in Case 1 could increase the total Coulomb stress by ~0.25 MPa compared to by ~0.12 MPa in Case 3 with single well operation. Therefore, adequate well design with respect to site-specific geological parameters (e.g., fault orientation) constrained during or prior to well operations can mitigate the earthquake nucleation driven by injection-extraction of fluids.

### Magnitude of induced earthquakes after shut-in

The maximum magnitude of earthquakes induced by subsurface energy exploration could be bounded by elastic strain energy and elevated pore pressure along the seismogenic fault plane. Based on a theoretical scaling relation between the maximum earthquake magnitude and the total injected volume (Δ*V*, proportional to $\Delta p$)^35^, a number of induced earthquakes with ${M}_{\text{w}}\ge 3.0$ predominantly associated with wastewater disposal observed in Oklahoma, USA^36^ are well correlated with Δ*V* whereas many large-magnitude earthquakes associated with hydraulic stimulation observed in West Canada Sedimentary Basin (WCSB) are not^30^. The presence of the fault plane in a critical state of stress may control the maximum magnitude^30^ and hydraulic fracturing operations can cause stress changes to activate fault slip at a distance of ≥1 km, while pore pressure accumulation inside a fault yields relatively long-term episodic seismicity^37^.

For the Pohang *M*_w_5.5 event, $\Delta V$ required to induce the earthquake was estimated to be higher by three orders of magnitude than the actual amount injected at the Pohang EGS site^26^. This substantial discrepancy highlights that observed larger magnitude earthquakes than expected should involve additional physical mechanisms enhancing accumulation and subsequent release of elastic energy. Previous studies of basin-scale Coulomb stress modeling indicated that successive earthquakes driven by tectonics could attribute to the stored strain energy along nearby faults (e.g., stress transfer and subsequent accumulation due to 2016 *M*_w_5.5 Gyeongju earthquake^25,38^). These studies suggest that even a slight increase in static stress may enhance the potential of earthquake nucleation along critically stressed faults located in the area under subsurface energy activities.

Furthermore, local perturbations in pore pressure and stress fields nearby the seismogenic fault plane could increase the scale of ${M}_{\text{w}}$ dramatically depending on site-specific factors, such as the well operation (e.g., larger rate, longer periods, and well design) and/or the formation characteristics (e.g., hydrological and mechanical properties, size of faults orientated favorable to slip, and/or the presence of hydraulic pathways permitting rapid pressure buildup)^39^. As shown in our study, multiple-well operation can enhance coupled poroelastic stressing and pore-pressure diffusion through tight and low permeability basement rocks that can accumulate strain energy on the fault even after all stimulations are over. Therefore, combined effects of regional tectonics and local stress perturbation driven by site-specific operational and geological features can enhance the cumulative moment along the fault, which supports small *b*-value of 0.7 ± 0.1 evaluated for the Pohang site^28,40^. Since the volume-based approach could be limited to estimate the potential risk of induced seismicity in the presence of hydraulically isolated fault(s), the risk mitigation with injection-extraction at multiple wells requires more robust physics-based analysis to predict a large earthquake in various geological systems.

## Conclusions

Our conceptual model of the sequential stimulation activities at the Pohang site shows that continuous pore-pressure diffusion and poroelastic shearing can bring about accumulation of substantial energy on the fault, potentially inducing moderate to large earthquakes even after shut-in. Poroelastic stressing can promote the activation of distant faults that are close to failure without requiring a direct hydraulic connection. Multiple well operations generate strong hydraulic gradients across the fault that can accelerate pore-pressure diffusion and elastic stress transfer into the fault. The low-permeability basement will delay pressure propagation to the hydraulically isolated fault, but can entrap elevated pore pressure within the fault. The less permeable and more rigid fault stores more energy, imposing higher probability to nucleate earthquakes at given stimulation operations. Therefore, site-specific operational and geological factors can enhance (or attenuate) the seismogenic response to the stimulation activities, and the local perturbation in stress states on the fault may be an additional critical mechanism to induce larger post shut-in earthquakes than theoretically expected. The findings of this mechanistic study suggest that comprehensive characterization of the faulting system and optimal well operation strategies are critical to mitigate potential seismic hazards associated with massive injection-extraction of fluids.

## Material and Methods

### Pohang geothermal stimulation site

Injecting high-pressure cold water into almost impermeable hot rocks generates permeable pathways by creating fractures or re-opening preexisting ones to exploit geothermal resources at a few kilometers of depth^41^. At the Pohang site in South Korea, the first EGS stimulation began on 29 January 2016 and a total of five phases of injection-production operations had taken place at ~4.3 km of depth granodioritic basement through PX-1 and PX-2 wells until September 2017 with a net injected volume of 6,000 m^3^ (total injected volume of 12,800 m^3^ and total extracted volume of 6,800 m^3^, Fig. 1 ^26,28,42^). The lack of seismicity in the area prior to the EGS operation and the proximity of the 2017 Pohang earthquake to an EGS site strongly support the feasibility of labeling the Pohang earthquake as a human-induced event^28^.

The spatial footprint of detected seismic events delineates the geometry of the fault plane (strike/dip = N214°/43°NW), separating PX-1 and PX-2^28,42^, which was not found prior to the EGS stimulation. The focal mechanisms indicate that the Korean Peninsula is under tectonic compression, and the local stress field reveals that the 2017 Pohang earthquake was induced by the oblique reverse slip of a previously extensional fault at optimal orientation^28^. The abrupt resurgence of seismicity releasing elastic strain during each stimulation phase indicates that the preexisitng fault is very sensitive to stress perturbations^28^. Accordingly, it is reasonable to assume critically-stressed condition on the fault, implying that the fault slipped with a small stress perturbation, and drilling or fluid injection-extraction initiated seismic activities along the fault^7^.

The hydraulic diffusivity for the basement ranges from $1\times {10}^{-4}$ to $1\times {10}^{-1}$ m^2^/s within the measured values of hydraulic conductivity ${K}_{b}={\kappa }_{b}{\rho }_{w}g/\eta$ (m/s), defined by the permeability ${\kappa }_{b}$ (m^2^) and the fluid viscosity $\eta$ (Pa$\cdot$s), and volumetric specific storage ${S}_{s,b}={\rho }_{w}g({c}_{r}+{\phi }_{b}{c}_{w})$ (1/m), where ${c}_{r}$ (1/Pa) and ${c}_{w}$ (1/Pa) are compressibilities for rock and fluid, respectively (refer to Fig. 6–1 and Section 6.2.1. in^28^). The detection of mud-loss in PX-2 at the depth of $3830 \sim 3840$ m where a fault zone was encountered suggests that the fault is hydraulically conductive^28^. Thus, the intermediate value for intensively fractured fault zones (ranges from $1\times {10}^{-14}$ to $1\times {10}^{-16}$ m^2^ ^43^) is assigned for the fault permeability.

### Numerical model setting

We model the three-dimensional (3-D) domain that represents the basement at a depth of 2 km including a mainshock fault, having an orientation of N214°/43°NW (Fig. 1B ^28,42^). The fault plane is modeled with a geometry of 0.9 km (L) × 1.5 km (H) × 0.005 km (W), approximated by the spatial distribution of seismic events including the *M*_w_5.5 earthquake^28,42^. The laterally extensive geometry (4 km length) is employed to minimize the boundary effects caused by diffusion. The finite-element analysis is performed using COMSOL Multiphysics 5.4^44^. A variable step method is employed for time integration^45^, and tetrahedral/cubic elements are used for spatial discretization^46^. Two separated sections are assigned for the surrounding basement to enhance numerical efficiency and accuracy: finer tetrahedral mesh within the inside cubic region whereas coarse tetrahedral mesh for the outer region (Fig. S1). Mesh was highly refined near the boundaries of the fault and the points for injection-extraction to resolve the strong pressure gradients driven by the contrast of material properties.

In the coupled system, the changes of the fluid content in pores perturb the pore-pressure field, and also deform the volume of the rock matrix, including the pore space, causing additional stresses. This process is explained by the theory of poroelasticity^47^, in which the flow variable (fluid pressure *p*) and mechanical response (displacement field **u**) are calculated simultaneously through a system of equations as follows^47–50^:

1 $${S}_{i}\dot{p}-\nabla \cdot {\Lambda }_{i}\nabla p+{\alpha }_{i}\nabla \cdot \dot{{\bf{u}}}=0,$$

2 $$\nabla ({\lambda }_{i}+{G}_{i})\nabla \cdot {\bf{u}}+\nabla \cdot {G}_{i}\nabla {\bf{u}}-{\alpha }_{i}\nabla p={\bf{r}},$$

where *S*_*i*_ (Pa^−1^) is the specific storativity and ${\lambda }_{i}$ (Pa) and *G*_*i*_ (Pa) are the Lam*é* elastic parameters, and ${\alpha }_{i}$ (−) is the Biot-Willis coefficient representing the ratio of changes in the fluid volume to the total bulk volume for deformation at constant pore pressure. ${\Lambda }_{i}\equiv {\kappa }_{i}/\eta$ is the flow mobility, where ${\kappa }_{i}$ (m^2^) is permeability and $\eta$ (Pa$\cdot$s) is fluid viscosity. The subscript *i* represents each material: basement ($b$), fault ($f$), and fluid ($w$), respectively. The source term **r** is a body force per unit bulk volume. Note that full poroelastic coupling is defined by the presence of $\nabla p$ in the force balance Eq. (2), acting as body forces in the stress equilibrium, and $\nabla \cdot {\bf{u}}$ in the flow Eq. (1). The hydrogeological and mechanical parameter values for Case 1 (reference model) are given in Table 1.

Solving the transient diffusion equation independently of the stress field reduces to the uncoupled system, widely used in hydrological model, as follows:

3 $${S}_{u,i}\dot{p}-\nabla \cdot {\Lambda }_{i}\nabla p=0,$$

where ${S}_{u,i}$ (Pa^−1^) is the uniaxial specific storativity defined under the conditions of uniaxial strain (${\varepsilon }_{11}={\varepsilon }_{22}=0$) and constant vertical stress (${\sigma }_{33}=c$). The hydraulic diffusivity can be expressed in terms of poroelastic coefficients as follows:

4 $${D}_{i}={\Lambda }_{i}\frac{({\lambda }_{u,i}-{\lambda }_{i})({\lambda }_{i}+2{G}_{i})}{{\alpha }_{i}^{2}({\lambda }_{u,i}+2{G}_{i})}=\frac{{\Lambda }_{i}}{{S}_{u,i}}.$$

The *in-situ* distributions of pressure and stress fields are not homogeneous and isotropic, such that poroelastic coupling could generate directional dependent changes in pressure and stresses. Therefore, this study implements the Coulomb stress change (Δ*τ*) from the initial state with assuming critically-stressed faults. The effects of poroelastic stressing and pore-pressure diffusion on Δ*τ* are evaluated using two terms: pore pressure change ($f\Delta p$) and the sum of the shear and normal stress components ($\Delta {\tau }_{s}+f\Delta {\sigma }_{n}$) where *f* is the fault friction coefficient. The uncoupled system used in the hydrological approach perturbs pore-pressure fields only, such that $\Delta \tau =f\Delta p$. This mechanistic study focuses on the perturbations in Δ*τ* from the equilibrium state that allows initial pore pressure and stresses set to zero. A constant pressure condition (*p* = 0) is hydraulically imposed on all boundaries which are free to move in the surface-parallel direction mechanically. Note that the hydrological model implements initial and boundary conditions only as a function of pore pressure.

The poroelastic response to each stimulation phase at either side of the fault determines stress components in space acting on the fault plane. Thus, we distinguished stimulation activities by PX-1 and PX-2 operations, respectively, not merely by combined injection and extraction volume changes as done in hydrological modeling approaches (Fig. 1C). Fluids are injected and extracted according to given well operation history, and the models are run for 750 days to analyze post shut-in response.

### Model case description

A series of sensitivity tests are performed to evaluate the influence of coupled mechanisms on the spatio-temporal patterns of Coulomb stress changes depending on operational and geological constraints. The parameters for each sensitivity test are given in Table 2. For the reference case (Case 1), we run a poroelasticity model with geological and operational information obtained from the Pohang EGS site. Comparison of Coulomb stress distributions from poroelasticity (coupled model, Case 1) and hydrological (uncoupled model, Case 2) models reveals the physical mechanism of coupled processes inducing post shut-in large-magnitude seismic events.

A better understanding of the driving mechanisms underlying the Pohang earthquake occurrence requires a re-examination of the operational controls on induced seismicity along hidden pre-existing faults. A simultaneous or sequential operation of injection-extraction through multiple wells has been proposed as a mitigation strategy to minimize geomechanical failure of the target formation by maintaining pore-pressure fields below the threshold for fault slip based on a mass balance approach^51,52^. Both the number of wells and the well locations with respect to the fault plane are the most essential parameters controlling earthquakes to limit the seismic hazards posed by given injection-extraction scenarios. Few studies, however, have focused on the local accumulation of pore pressure and elastic strain formed by wells and preexisting faults acting as hydraulic/mechanical conduits or barriers. We conducted a coupled simulation with a single-well operation in which whole injection-extraction activities were operated only through PX-2 (Fig. 4B). Note that a single-well operation setting only aims to look into how well design with respect to the fault geometry influences the mechanical stability of preexisting weak structures, not considering the operational efficacy for a heat exchanger.

The onset and subsequent occurrence of induce seismicity are determined by hydrological and mechanical properties of a fault zone and surrounding basement rocks. However, the limit of field-based data acquisition as well as intrinsic complexity of a fault zone, driven by internal architecture, host rock lithology, and/or tectonics, hinder precise characterization of its properties^53^. From the experimental and field data, the overall fault permeability (${\kappa }_{f}$) and shear modulus (*G*_*f*_) are estimated to range from 10^−12^ to 10^−22^ m^2^ ^53^ and from 0.8 to 20 GPa^43^, respectively, depending on the internal architecture of the fault. For the sensitivity test, we analyze how the end-member (largest) parameter values for basement permeability or fault properties, representing a highly permeable or more rigid fault zone, affect pore-pressure and stress fields along the fault.

## Supplementary information

Supplementary Information.
