# Supplementary material for: Operational and geological controls of coupled poroelastic stressing and pore-pressure accumulation along faults: Induced earthquakes in Pohang, South Korea
Source: Sci Rep. 2020 Feb 7;10:2073. doi: 10.1038/s41598-020-58881-z (PMC7005858; doi:10.1038/s41598-020-58881-z)
Supplement: Supplementary file 1 — Supplementary Information. [file 41598_2020_58881_MOESM1_ESM.pdf]

Supplementary Materials

Title: Operational and geological controls of coupled poroelastic stressing and pore-pressure accumulation along faults: Induced earthquakes in Pohang, South Korea.

Kyung Won Chang[1], Hongkyu Yoon[2], YoungHee Kim[3], Moo Yul Lee[2].

1 Sandia National Laboratories, Geotechnology & Engineering Department, Albuquerque, 87105, USA.

2 Sandia National Laboratories, Geomechanics Department, Albuquerque, 87105, USA.

3 Seoul National University, School of Earth and Environmental Sciences, South Korea.

Finite element simulation setting

In COMSOL Multiphysics, the "Poroelasticity" interface couples Darcy's law and solid mechanics to model deformation of porous media caused by fluid injection-extraction. The overall procedure is as follows:

1. Select model dimension (3-D)
2. Select governing physics interfaces (**Darcy's Law** and **Solid Mechanics**)
3. Add multiphysics interface **Poroelasticity**
4. Import well operation data (.txt format)
5. Generate geometry
6. Define hydrological and mechanical parameter values
7. Select **Poroelastic Storage** for Darcy's law
8. Define initial and boundary conditions for each physics interface
9. Locate fluid and matrix properties for each physics interface
10. Generate mesh
11. Define time steps for simulation and results
12. Run simulation
13. Visualize and export results for the Coulomb stress analysis

In our three-dimensional (3-D) finite element model, two separated sections are assigned for the surrounding basement to enhance numerical efficiency: finer tetrahedral mesh within the inside cubic region whereas coarse tetrahedral mesh for the outer region (Figure S1). Fine mapped mesh is implemented in the fault region to achieve better accuracy in mechanical solutions. Tetrahedral mesh is highly refined near the boundaries of the fault and the points for injection-extraction to resolve the strong pressure gradients caused by the contrast of material properties.

Hydrological model results with variation in basement permeability

The hydrological modeling approach has focused on the direct pore-pressure impact on the fault slip and suggested the enlargement of pressurized regions encountering the locked fault as a critical factor to induce earthquakes. A recent hydrological model tried to match stimulation history (Figure S2A) and the temporal evolution of seismic events observed at Pohang by setting the hydraulic diffusivity of the basement rock surrounding the fault plane to 1×10^{-2} m^2/s (converted to permeability $\kappa_b = 7.6 \times 10^{-17}$ m^2) that was the practical upper limit of the hydraulic diffusivity estimated from hydraulic modeling calibration and analytical Jacob method (measured values within $1 \times 10^{-4} \leq D_b \leq 1 \times 10^{-1}$ m^2/s; refer to Fig. 6-1 and Section 6.2.1. in[1]). Our uncoupled hydrological model results show that moderate to large earthquakes, including the M_W5.5 event, are unlikely to occur due to substantial dissipation of elevated pore pressure into the high-diffusivity basement after extraction or shut-in. (solid line in Figure S2B).

This pore-pressure behavior is similar to one obtained from the numerical model used for the Korean Government Commission Report[1] which also shows rapid pore-pressure buildup with stimulation and following attenuation after terminating stimulation (Figure S3, reproduction of Figure O-15b given in the Report[1]). Note that the hydrological model in[1] implemented two fault planes located at the hypocenters of M_W3.2 and M_W5.5 earthquakes. The presence of high-permeability fault plane nearby the M_W3.2 event causes less pore-pressure buildup after the second stimulation through PX-1 compared to the result from this study shown in Figures 3D and S2B.

Under a diffusion-dominant system, the pore-pressure buildup within the permeable fault is essential to nucleate large-magnitude earthquakes, which is controlled by a contrast of hydraulic diffusivity between a fault and a bounding

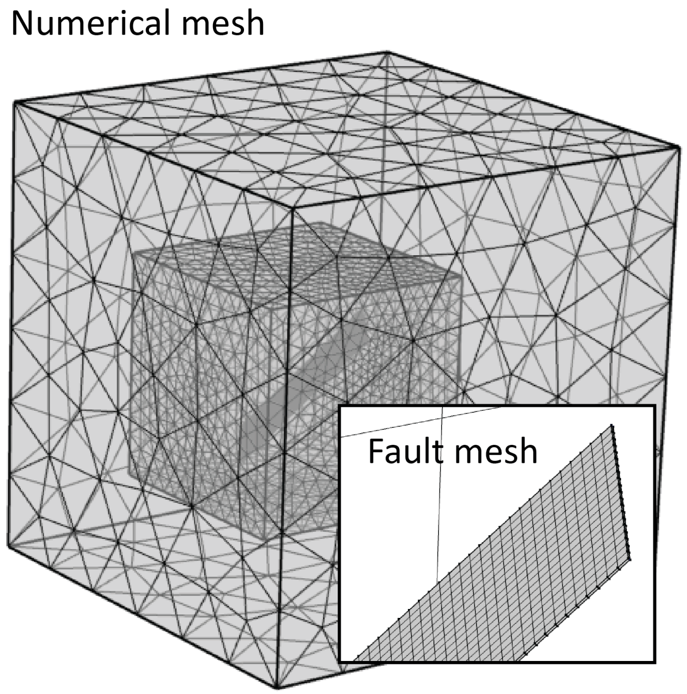

Figure S1. Cubic meshes are implemented in the fault whereas tetrahedral meshes are used in the surrounding basement. Two regions are assigned for the surrounding domain to save the numerical costs by using coarser tetrahedral meshes in the outer region.

basement. Less permeable basement rocks will decelerate diffusion of pore pressure into the fault, but enhance the efficacy of trapping accumulated pore pressure within the fault after shut-in. Reducing an order of magnitude of the basement permeability ($\kappa_b = 7.6 \times 10^{-18}$ m^2) fails to generate immediate pore-pressure accumulation within the fault, such that pore-pressure evolution at the hypocenter is not in accord with the initial seismic events (dashed line in Figure S2B). Seismic events afterwards cannot be described by this model due to no substantial increases in pore pressure despite stimulation Phase 3 (injection through PX-2 around $\Delta t = 450$ days) as well as no accumulation of pore pressure after shut-in ($\Delta t \geq 645$ days).

Changing the basement permeability to $\kappa_b = 7.6 \times 10^{-19}$ m^2/s generates monotonic increases of pore pressure at the Pohang earthquake hypocenter after the second injection operation (dotted line in Figure S2B). The low-diffusivity basement traps elevated pore-pressure within the fault zone, which may induce the post shut-in M_{W} 5.5 earthquake. Notwithstanding, the postponed diffusion process fails to capture the seismic events during and after Phases 1 and 2 stimulation. The inconsistency between temporal patterns of pore-pressure changes from conventional hydrological modeling and consecutive occurrence of earthquakes addresses that the Pohang earthquake was most likely to involve additional physical mechanisms.

Enhancement of coupling effects by operational constraints

The operation of multiple wells can generate gradients in pressure and stress fields, which determines directional characteristics of diffusion and poroelastic stressing. Figure S4 shows how significantly well operation influences the coupled process of pore-pressure diffusion and poroelastic stressing. The single-well model shows that almost no critical impact of pore-pressure diffusion on the fault (Figure S4B) because cyclic injection-extraction operations through PX-2 causes weak gradients in pore-pressure fields that inhibits the pore-pressure dissipation into the fault (refer to Figures 4A and 4B describing mechanism schematically). Instead, shear stressing as poroelastic response to compression plays a main role in positive increases of $\Delta\tau$ corresponding to the sequential well operations (Figures S4D to S4F).

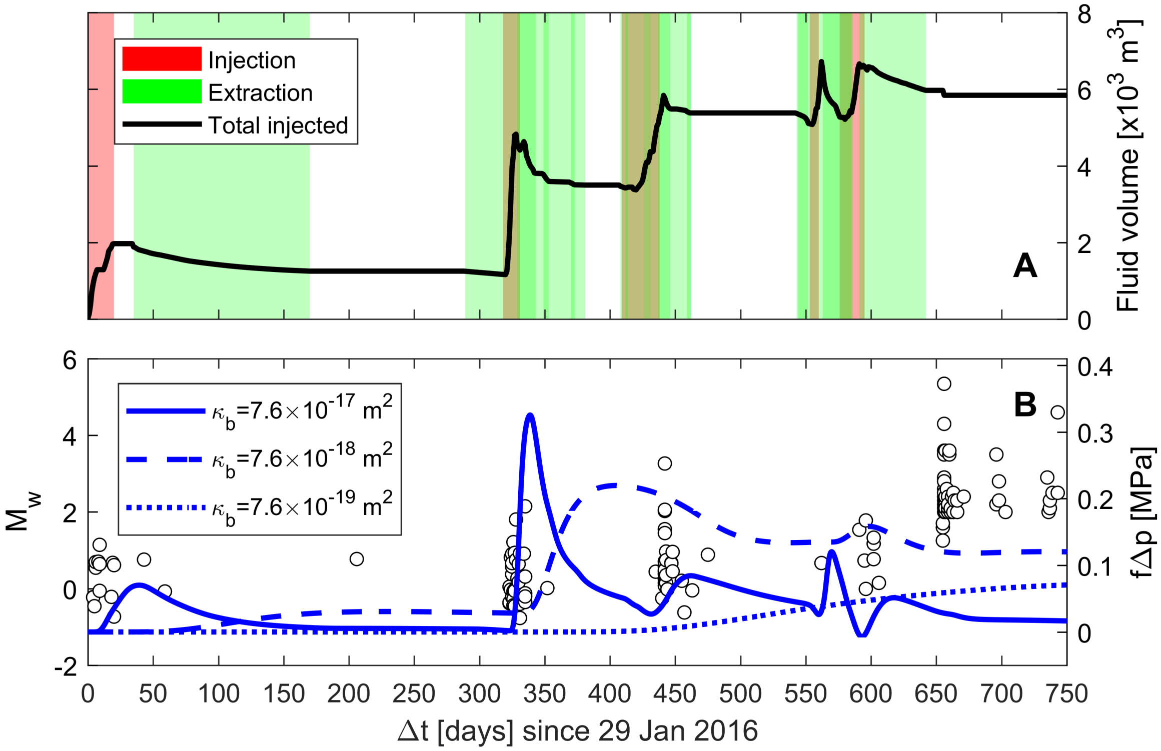

Figure S2. Hydrological model results. A. Injection-production history at the Pohang EGS site[1–3]. The accumulative injection volume is indicated by a black solid line, and injection and extraction operations are indicated by red and green bars, respectively. B. The temporal evolution of pore-pressure perturbations ($f\Delta p$) at the fault plane from hydrological models with variation in basement permeability (κ_b)

Poroelasticity model results with variation in basement permeability

From all simulations with the variation in geological and operational settings, we obtain the temporal changes in pore pressure, poroelastic stresses, and Coulomb stress fields along the middle of the fault plane. The vertical axis of each plot is from the fault top ($z = -3.6$ km) whereas the lower bound is the fault bottom ($z = -4.6$ km). Figure S5 shows the spatio-temporal distribution of Coulomb stress components along the fault middle: reference model results in the left column and high-permeability basement case in the right column.

Low-permeability basements in the reference model limit pore-pressure propagation, such that no changes in $f\Delta p$ is observed after the Phase 1 stimulation (Figure S5A). The initiation of the Phase 2 stimulation, injecting a large amount of fluids at PX-1 while extraction at PX-2 simultaneously, causes substantial compression on the fault that increases $f\Delta p$ as poroelastic response. On the other hand, high-permeabiity basements allows rapid diffusion of pore pressure into the fault (Figure S5B). A spatial and temporal pattern of positive $\Delta\tau_s + f\Delta\sigma_n$ represents compression acting on the fault, driven by the sequential stimulation activities at both sides of the fault (Figures S5C and S5D), which is consistent to the focal mechanisms and stress models showing that the fault was reactivated mainly by reverse slip[1]. The summation of pore pressure and normal and shear stresses gives $\Delta\tau$, and the spatio-temporal patterns are formed corresponding to the stimulation location and history (Figure S5E). However, rapid dissipation of pore pressure back into the high-permeability basement cannot accumulate energy enough to cause large-magnitude earthquake after shut-in (Figure S5F).

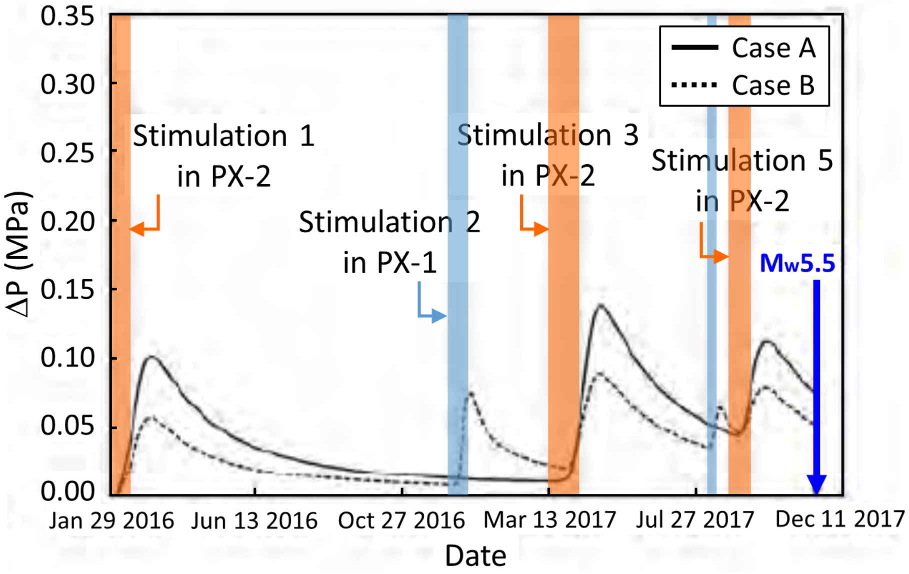

Figure S3. Reproduction of Figure O-15b for hydrological model results from the Korean Government Commission Report[1]. Pore-pressure changes at the hypocenters of M_W5.5 seismic events: Case A for the mainshock fault (which has similar geometry and orientation with the fault modeled in this study) with a low-permeability core whereas Case B for the mainshock fault not including a low-permeability core. The rapid response of pore-pressure buildup/attenuation with each stimulation activity cannot support the occurrence of M_W5.5 earthquake after shut-in of all wells.

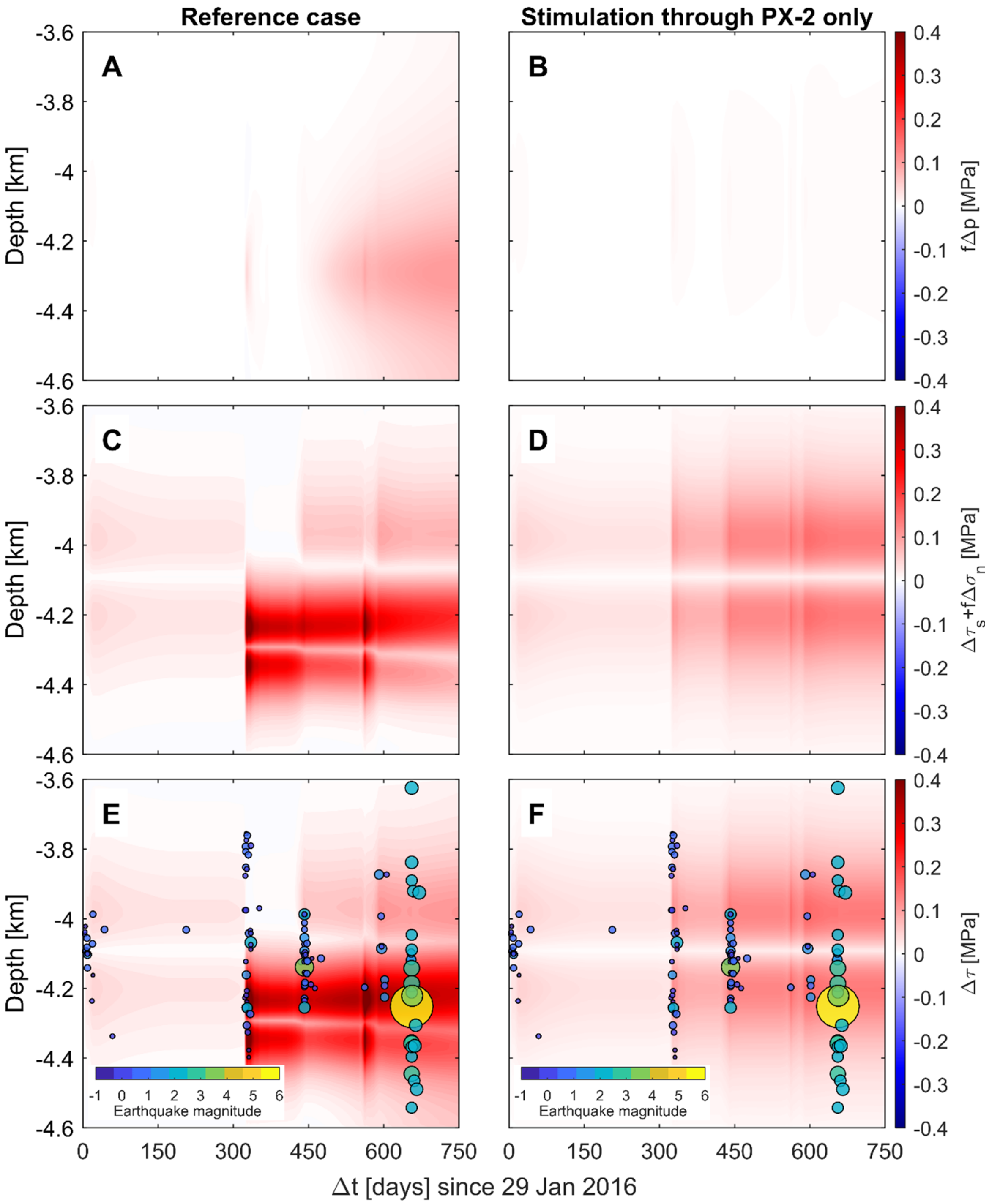

Figure S4. Spatio-temporal distributions of the Coulomb stress components $f\Delta p$ (A–B), $\Delta\tau_s + f\Delta\sigma_n$ (C–D), and $\Delta\tau$ (E–F) along the fault middle with two different well design: alternate injection-extraction through PX-1 and PX-2 (Case 1; left column) and injection-extraction only through PX-2 (Case 3; right column).

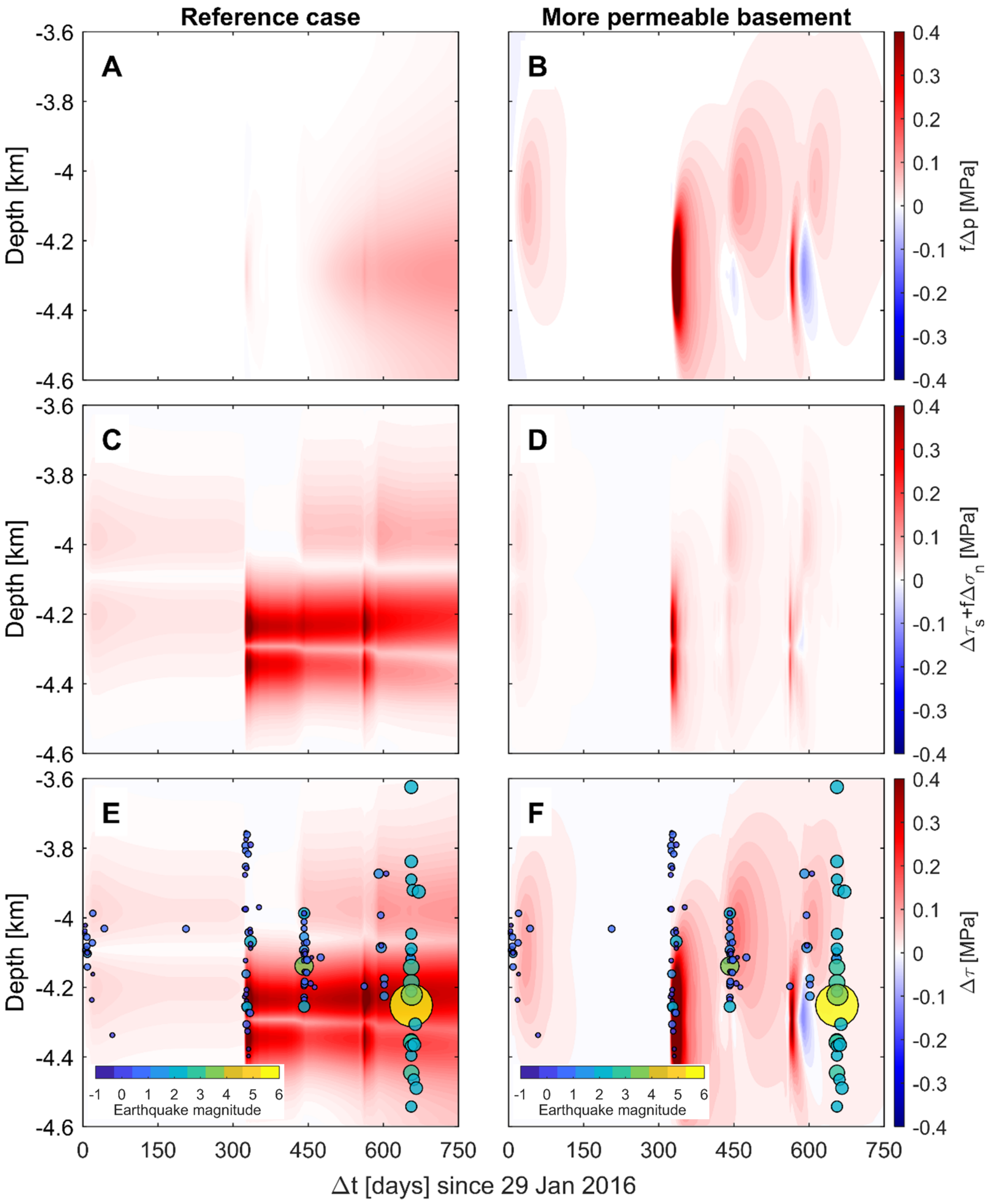

Figure S5. Spatio-temporal distributions of the Coulomb stress components $f\Delta p$ (A–B), $\Delta\tau_s + f\Delta\sigma_n$ (C–D), and $\Delta\tau$ (E–F) along the fault middle with variation in basement permeability. Results from Case 4 simulation (more permeable basement) are shown in the right column) .

References

1. Geological Society of Korea. *Final Report of the Korean Government Commission on Relations between the 2017 Pohang Earthquake and EGS Project* (2019).

2. Ellsworth, W. L., Giardini, D., Townend, J., Ge, S. & Shimamoto, T. Triggering of the Pohang, Korea, Earthquake (M_W 5.5) by Enhanced Geothermal System Stimulation. *Seism. Res. Lett.* **90**, 1844–1858, DOI: 10.1785/0220190102 (2019).

3. Kim, K.-H. *et al.* Assessing whether the 2017 M_w 5.4 Pohang earthquake in South Korea was an induced event. *Science* **360**, 1007–1009, DOI: 10.1126/science.aat6081 (2018).

Supplementary materials

Supplementary Text
Figures S1 to S5
References *(1-3)*
